# Gallbladder small cell carcinoma: a case report and literature review

**DOI:** 10.1186/s40792-016-0200-3

**Published:** 2016-07-25

**Authors:** Toshiyuki Adachi, Masashi Haraguchi, Junji Irie, Tomoko Yoshimoto, Ryohei Uehara, Shinichiro Ito, Hirotaka Tokai, Kazumasa Noda, Nobuhiro Tada, Masataka Hirabaru, Keiji Inoue, Shigeki Minami, Susumu Eguchi

**Affiliations:** 1 Department of Surgery, Nagasaki Harbor Medical Center City Hospital, Nagasaki, 850-8555 Japan; 2 Department of Pathology, Nagasaki Harbor Medical Center City Hospital, Nagasaki, 850-8555 Japan; 3 Department of Internal Medicine, Nagasaki Harbor Medical Center City Hospital, Nagasaki, 850-8555 Japan; 4 Department of Surgery, Nagasaki University Graduate School of Biomedical Sciences, Nagasaki, 852-8501 Japan

**Keywords:** Small cell carcinoma, Gallbladder, Neuroendocrine tumor

## Abstract

Gallbladder small cell carcinoma (SCC) comprises only 0.5 % of all gallbladder cancer and consists of aggressive tumors with poor survival outcomes against current treatments. These tumors are most common in elderly females, particularly those with cholecystolithiasis. We report the case of a 79-year-old woman with gallbladder small cell carcinoma. The patient had intermittent right upper quadrant abdominal pain and was admitted to our hospital due to suspected acute cholecystitis. She regularly received medical treatment for diabetes, hypertension, and dyslipidemia. On initial laboratory evaluation, the levels of aspartate aminotransferase (AST), total bilirubin, and C-reactive protein (CRP) were markedly elevated. She underwent computed tomography (CT) for screening. CT images showed a thick-walled gallbladder containing multiple stones and multiple 3-cm-sized round nodular lesions, which were suggestive of metastatic lymph nodes. After percutaneous transhepatic gallbladder drainage was performed, endoscopic ultrasound-guided fine needle aspiration of enlarged lymph nodes resulted in a diagnosis of small cell carcinoma or adenocarcinoma. However, we could not identify the primary lesion before the surgery because of no decisive factors. We performed cholecystectomy because there was a possibility of cholecystitis recurrence risk and also partial liver resection because we suspected tumor invasion. The final pathological diagnosis was neuroendocrine carcinoma of the gallbladder, small cell type. The tumor stage was IVb, T3aN1M1. The patient died 13 weeks after the surgery. In the present paper, we review the current available English-language literature of gallbladder SCC.

## Background

Primary gallbladder neuroendocrine tumors are rare, representing 0.2 % of all tumors [1]. Neuroendocrine neoplasms of the gallbladder are classified as grades 1 and 2 neuroendocrine tumor (NET), neuroendocrine carcinoma (NEC) (large cell or small cell type), and mixed adenoneuroendocrine carcinoma (MANEC) [2]. In particular, gallbladder small cell carcinoma (SCC) is extremely rare. Gallbladder SCC carries a grave prognosis with the survival rates worse than gallbladder adenocarcinoma due to its high malignant potential and late stage at presentation [2]. The overall median survival is 4–6 months despite aggressive management. According to the Surveillance, Epidemiology and End Results (SEER) data, the 1-year survival of gallbladder SCC was 21 % and 5-year was 0 % [3]. In recent years, gallbladder SCC has attracted increasing attention for improved understanding and diagnosis. Herein, we describe the latest review of the English-language literature, including our present gallbladder SCC case [2–17].

## Case presentation

**Fig. 1:**
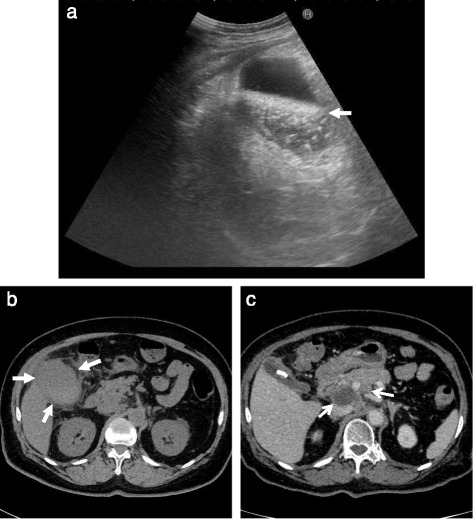
Ultrasonographic finding. **a** The gallbladder was filled with many small stones and sludge (*white solid arrow*). Computed tomographic findings. **b** Computed tomography (CT) showing a thick-walled and enlarged gallbladder (*white solid arrows*). **c** After percutaneous transhepatic gallbladder drainage, contrast-enhanced CT showing multiple round nodular lesions, which were suggestive of metastatic lymph nodes (*white arrows*)

**Fig. 2:**
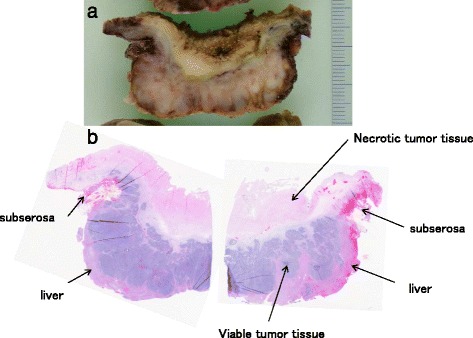
Gallbladder tumor. **a** Appearance of gallbladder tumor section. **b** Microscopic finding showing necrotic tissue of gallbladder. The layer of mucosa and muscularis propria layers of the gallbladder were not seen because of tumor proliferation with marked necrosis (*panoramic view*)

**Fig. 3:**
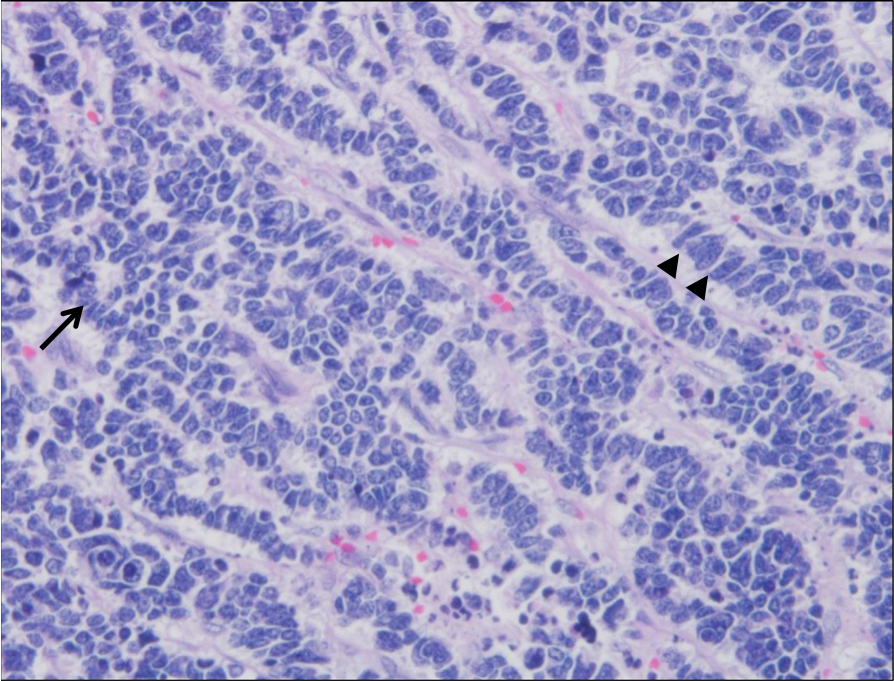
Histological examination of gallbladder tumor. The cellularity is very high with hyperchromatic nuclei with scant cytoplasm. Rosette forming (*black arrow*) and nuclear molding (*black arrow heads*) (H&E stain, ×400)

**Fig. 4:**
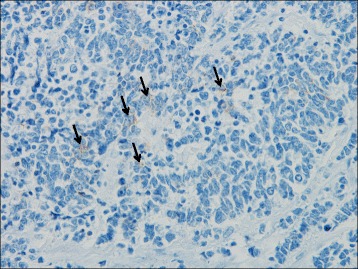
Immunohistochemical staining for chromogaranin A showed focal staining (*black arrows*) (H&E stain, ×400)

A 79-year-old woman visited a local internal medicine clinic because of intermittent right upper quadrant abdominal pain. The patient was admitted to our hospital due to suspected acute cholecystitis and had Murphy’s sign on admission. The patient was being regularly treated for diabetes, hypertension, and dyslipidemia, but she did not smoke or drink alcohol. Additionally, the patient had a history of breast cancer diagnosed 22 years previously that had been treated with mastectomy. Routine laboratory evaluation showed white blood cell count at 10,100/μl, total bilirubin at 7.9 mg/dl, aspartate aminotransferase (AST) at 113 U/l, alanine aminotransferase (ALT) at 2 U/l, and C-reactive protein (CRP) at 30.38 mg/dl. We regrettably did not check tumor markers. The patient underwent ultrasonography (US) and computed tomography (CT), with and without contrast, for screening. The US images revealed many small stones and sludge in the gallbladder (Fig. 1a). CT images similarly revealed a thick-walled gallbladder containing multiple stones (Fig. 1b). Multiple 3-cm-sized round nodular lesions with heterogeneous enhancement were also noted in the peri-cholecystic and peri-pancreatic area, which were suggestive of metastatic lymph nodes (Fig. 1c). Percutaneous transhepatic gallbladder drainage was then performed, as an alternative interim strategy before surgery. Abdominal symptoms subsequently improved, and endoscopic ultrasound-guided fine needle aspiration of enlarged lymph nodes resulted in a diagnosis of small cell carcinoma or adenocarcinoma. However, we could not identify the primary lesion. Therefore, our surgical procedure included radical cholecystectomy with partial liver resection together with lymph node dissection. Pathology of the resected specimen showed that the entire gallbladder was tumorous. Additionally, the tumors invaded into the liver (Fig. 2a, b). Histopathological examination of the tumors revealed neuroendocrine carcinoma, small cell type (Fig. 3). The tumor stage of this patient was IVb, T3aN1M1. On immunohistochemical study, the tumor cells showed positive staining for chromogranin and negative staining for synaptophysin (Fig. 4). Postoperatively, the patient developed progressive jaundice unrelieved with biliary stenting and died 13 weeks after the surgery.

### Discussion

Neuroendocrine tumors (NETs) usually appear in the gastrointestinal and bronchopulmonary systems. There are no neuroectodermal cells in the gallbladder mucosa. Therefore, primary gallbladder neuroendocrine tumors are postulated to arise from either a multipotent stem cell or neroendocrine cells in intestinal or gastric metaplasia secondary to cholelithiasis and chronic cholecystitis [3]. In neuroendocrine neoplasms of the gallbladder, SCC is extremely rare: although 1002 cholecystectomy cases have been performed at our hospital since 1997, the case described was our first of gallbladder SCC. In recent years, gallbladder SCC has attracted increasing attention for improved understanding and diagnosis. As shown in Table 1 (compiled from our literature survey), the average age of diagnosis is 64 years, with a male-to-female ratio of 1:1.9. In most cases of gallbladder SCCs, patients typically present with recurrent right upper quadrant pain. Less frequent presentations include abdominal mass, weight loss, and ascites [16]. Gallbladder SCCs are commonly associated with stones, with cholelithiasis found in 49 % of cases (Table 1). A preoperative diagnosis of gallbladder SCC is very difficult because the presentation generally consists of nonspecific symptoms. And, despite the development of imaging studies, it is also still difficult to diagnose gallbladder SCC by ultrasonography, CT, or magnetic resonance imaging. Therefore, the diagnosis is often made incidentally at the time of cholecystectomy performed for cholecystolithiasis or polyps. Recent studies indicate that the role of 18F-FDG positron emission tomography (PET)/CT might prove to be useful in diagnostic imaging for the detection of gallbladder SCC [8]. However, we regrettably do not have a PET-CT scanner in our hospital.

**Table 1:** Characteristics of patients with gallbladder small cell carcinoma

Gender (*n* = 121)	
Men	42 (35 %)
Women	79 (65 %)
Median age in years (*n* = 120)	64
Histopathology (*n* = 104)	
Pure small cell carcinoma	82 (79 %)
Combined	22 (21 %)
Cholelithiasis (*n* = 120)	59 (49 %)
Treatment	
Surgery (*n* = 90)	67 (74 %)
Chemotherapy (*n* = 90)	53 (59 %)
Stage (*n* = 117)	
I–III	39 (33 %)
IV	78 (67 %)
Metastases	
Lymph nodes (*n* = 118)	83 (70 %)
Liver (*n* = 117)	66 (56 %)
Lung (*n* = 117)	7 (6 %)
Pancreas (*n* = 117)	7 (6 %)
Peritoneum (*n* = 117)	6 (5 %)
Omentum (*n* = 117)	5 (4 %)
Median survival in months	8

*n* number of patients for whom data were available for the particular characteristic

Gallbladder SCC usually presents as a large mass containing extensive necrosis with marked propensity for invasive submucosal growth [18]. Histopathologically, about 80 % of cases are pure SCC and the remaining 20 % are combined SCC (Table 1). Microscopically, the WHO classification defines small cell carcinomas as neuroendocrine tumors with >20 mitoses/2 mm^2^ (mean 75/10 hpf) and small cell cytological features [16]. Immunohistochemically, the tumor cells expressed neuroendocrine markers, such as chromogranin A, synaptophysin, and/or CD56. In comparison to NETs which usually are diffusely positive for neuroendocrine markers, SCCs show more focal staining [19]. Surgical treatment remains the best curative option and appears to prolong life, with chemotherapy adding a marginal advantage. The operative procedures that we deem appropriate range from cholecystectomy alone to extensive surgical resections, including regional lymph node clearances and hepatic lobectomy. However, therapeutic options are limited because the disease presents in an advanced stage. In fact, stage IV is found in 67 % of cases (Table 1). In cases of nonresectable tumors, the primary management is chemotherapy, and the chemotherapeutic agents of choice are cisplatin, etoposide, and 5-fluorouracil. Actually, there are few papers that showed specific chemotherapy regimens [2, 7]. Surgical treatment and chemotherapy are found in 74 and 59 % of cases, respectively (Table 1). The role of radiotherapy remains undefined due to paucity of data [2]. As shown in Table 1, distant metastasis at presentation is present and is most often located in adjacent lymph nodes (70 %) and the liver (56 %). Median survival for gallbladder SCC is only 8 months. Based on SEER data, gallbladder SCC has no survivors at 10 years, in contrast to the 10-year survival of 36 % for carcinoid tumors [18].

## Conclusions

We have presented the latest review of English-language literature concerning gallbladder SCC. However, despite the finding of a few recent useful papers, we could not give unequivocal directions on preoperative diagnostic methods and treatments including specific chemotherapy regimens. As with previous reports, we can assume that aggressive multimodal treatment may prolong survival of gallbladder SCC patients if they could be provided with early diagnosis with prompt surgical intervention. But, we do not have more effective targeted treatment modalities for gallbladder SCC as yet. Thus, prospective studies on a larger scale need to be conducted in the future.

## Consent

Written informed consent was obtained from the next of kin of the patient for publishing this case report with accompanying images because the patient had died. A copy of the written consent is available for review by the Editor-in-Chief of this journal.

## References

[1] Mezi S, Petrozza V, Schillaci O, La Torre V, Cimadon B, Leopizzi M (2011). Neuroendocrine tumors of the gallbladder: a case report and review of the literature. J Med Case Rep.

[2] Kamboj M, Gandhi JS, Gupta G, Sharma A, Pasricha S, Mehta A (2015). Neuroendocrine carcinoma of gall bladder: a series of 19 cases with review of literature. J Gastrointest Cancer.

[3] Eltawil KM, Gustafsson BI, Kidd M, Modlin IM (2010). Neuroendocrine tumors of the gallbladder: an evaluation and reassessment of management strategy. J Clin Gastroenterol.

[4] Mahipal A, Gupta S (2010). Small-cell carcinoma of the gallbladder: report of a case and literature review. Gastrointest Cancer Res.

[5] Matsuo S, Shinozaki T, Yamaguchi S, Matsuzaki S, Takami Y, Hayashi T (2000). Small-cell carcinoma of the gallbladder: report of a case. Surg Today.

[6] Iype S, Mirza TA, Propper DJ, Bhattacharya S, Feakins RM, Kocher HM (2009). Neuroendocrine tumours of the gallbladder: three cases and a review of the literature. Postgrad Med J.

[7] Usmani S, Pazooki M, Bilgrami SF (2010). Small cell carcinoma of the gall bladder: role of adjuvant chemotherapy. J Gastrointest Cancer.

[8] Kim DM, Yang SO, Han HY, Kim KS, Son HJ (2010). Small cell carcinoma of the gallbladder: ^18^F-FDG PET/CT imaging features—a case report. Nucl Med Mol Imaging.

[9] Nau P, Liu J, Dillhoff M, Forster M, Hazey J, Melvin S (2010). Two cases of small cell carcinoma of the gallbladder. Case Rep Med.

[10] Lee JM, Hwang S, Lee SG, Lee YJ, Park KM, Kim KH (2010). Neuroendocrine tumors of the gallbladder: twelve cases in a single institution. Hepatogastroenterology.

[11] Furrukh M, Qureshi A, Saparamadu A, Kumar S (2013). Malignant neuroendocrine tumour of the gallbladder with elevated carcinoembryonic antigen: case report and literature review. BMJ Case Rep.

[12] Tamura T, Takeuchi K (2013). Small cell gall bladder carcinoma complicated by syndrome of inappropriate secretion of antidiuretic hormone (SIADH) treated with mozavaptan. BMJ Case Rep.

[13] Tunio MA, Alasiri M, Ali AM, Alsaeed EF, Shuja M, Fatani H (2013). Distal humerus as delayed site of metastasis from small cell carcinoma of gallbladder. Case Rep Gastrointest Med.

[14] Aiello P, Aragona F, Territo V, Caruso AM, Patti R, Buscemi S (2014). Concomitant small cell neuroendocrine carcinoma of gallbladder and breast cancer. Case Rep Surg.

[15] Yun SP, Shin N, Seo HI (2015). Clinical outcomes of small cell neuroendocrine carcinoma and adenocarcinoma of the gallbladder. World J Gastroenterol.

[16] Nemenqani DM, Fuloria J, Karam RA, Hammadi H (2015). Gallbladder neuroendocrine neoplasms: a case report of gallbladder small cell carcinoma. J Gastrointest Cancer.

[17] Chen C, Wang L, Liu X, Zhang G, Zhao Y, Geng Z (2015). Gallbladder neuroendocrine carcinoma: report of 10 cases and comparison of clinicopathologic features with gallbladder adenocarcinoma. Int J Clin Exp Pathol.

[18] Kanthan R, Senger JL, Ahmed S, Kanthan SC (2015). Gallbladder cancer in the 21st century. J Oncol.

[19] Maitra A, Tascilar M, Hruban RH, Offerhaus GJ, Albores-Saavedra J (2001). Small cell carcinoma of the gallbladder: a clinicopathologic, immunohistochemical, and molecular pathology study of 12 cases. Am J Surg Pathol.

